# A retrospective real-world study of early short-course remdesivir in non-hospitalized COVID-19 patients at high risk for progression: low rate of hospitalization or death, regardless of immunocompetence status

**DOI:** 10.3389/fphar.2023.1218650

**Published:** 2023-10-10

**Authors:** José Manuel Ramos-Rincón, Héctor Pinargote-Celorio, Jara Llenas-García, Oscar Moreno-Pérez, Inmaculada González-Cuello, Pilar Gonzalez-de-la-Aleja, Belén Martínez-López, Sergio Reus, María García-López, Juan Carlos Rodríguez, Vicente Boix, Esperanza Merino

**Affiliations:** ^1^ Internal Medicine Department, Dr. Balmis General University Hospital—Alicante Institute of Health and Biomedical Research (ISABIAL), Alicante, Spain; ^2^ Clinical Medicine Department, Miguel Hernández University, Elche, Spain; ^3^ Unit of Infectious Diseases Dr. Balmis General University Hospital—Alicante Institute of Health and Biomedical Research (ISABIAL), Alicante, Spain; ^4^ Internal Medicine Service Vega Baja Hospital—Foundation for the Promotion of Health and Biomedical Research of the Valencian Community (FISABIO), Valencia, Spain; ^5^ Endocrinology Department Dr. Balmis General University Hospital—Alicante Institute of Health and Biomedical Research (ISABIAL), Alicante, Spain; ^6^ Microbiology Service Dr. Balmis General University Hospital—Alicante Institute of Health and Biomedical Research (ISABIAL), Alicante, Spain

**Keywords:** SARS-CoV-2 early treatment, remdesivir, outpatient SARS-CoV-2 treatment, immunosupressed SARS-CoV2 treatment, hospitalization SARS-CoV-2 infection

## Abstract

**Introduction:** The evidence for remdesivir therapy in immunocompromised patients is scarce. To evaluate remdesivir (RDV) effectiveness and safety in COVID-19 outpatients at high risk for progression in a real-world setting, we compare the outcome in immunocompromised (IC) patients with that in non-immunocompromised patients.

**Methods:** Two hospitals conducted a retrospective study of all adult patients with mild-to-moderate SARS-CoV-2 infection at high risk for disease progression who were treated as outpatients with a 3-day course of RDV (1st January−30th September 2022). The primary effectiveness endpoint was a composite of any cause of hospitalization or death by day 30. A multiple logistic regression model was built to explore the association between immune status and clinical outcome, estimating adjusted odds ratios [aORs (95% CI)].

**Results:** We have included 211 patients, of which 57% were males, with a median age of 65 years (IQR 53–77), 70.1% were vaccinated (three or four doses), and 61.1% were IC. The median duration of symptoms before RDV treatment was 3 days (IQR 2–5). During follow-up, 14 (6.6%) patients were hospitalized, of which 6 (2.8%) were hospitalized for COVID-19 progression. No patient required mechanical ventilation, and two patients died (non-COVID-19-related). After accounting for potential confounders, only anti-CD20 treatment was associated with the composed outcome [aOR 5.35 (1.02–27.5, 95% CI)], whereas the immunocompetence status was not [aOR 1.94 (0.49–7.81, 95% CI)].

**Conclusion:** Early COVID-19 outpatient treatment with a 3-day course of remdesivir in vaccinated patients at high risk for disease progression during the Omicron surge had a good safety profile. It was associated with a low rate of all-cause hospitalization or death, regardless of immunocompetence status.

## Graphical Abstract

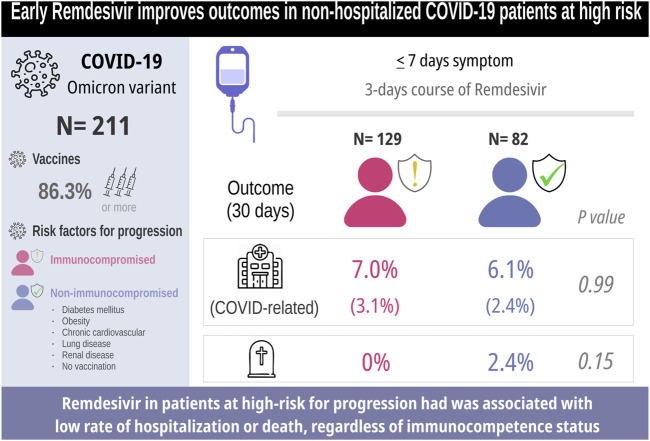

## 1 Introduction

Antiviral treatment [nirmatrelvir/ritonavir, molnupiravir, and remdesivir (RDV)] for non-hospitalized COVID-19 patients at high risk of progression was initially supported by clinical trials in unvaccinated people during the pre-Omicron wave (Gottlieb et al., 2022; Hammond et al., 2022; Jayk Bernal et al., 2022). Subsequently, three clinical trials among predominantly vaccinated outpatients during the Omicron wave have been reported, but no clinical benefit of molnupiravir was demonstrated (Butler et al., 2023), while a single dose of pegylated interferon lambda reduced the incidence of hospitalization or emergency department visit, and VV116 administration was superior to that of nirmatrelvir–ritonavir with respect to the time to sustained clinical recovery (Cao et al., 2023; Reis et al., 2023). Therefore, we currently have only one oral treatment drug, nirmatrelvir/ritonavir, which is effective in reducing the progression to severe COVID-19. However, immunocompromised patients are underrepresented in these trials. Recent observational cohorts report the effectiveness of nirmatrelvir/ritonavir in real-world settings, with 21%–79% reductions in the risk of COVID-19-related admission (Arbel et al., 2022; Najjar-Debbiny et al., 2022).

Remdesivir has been associated with reduction or progression in hospitalized patients who required no or conventional oxygen support, according to a recent meta-analysis (Amstuzt et al., 2023). However, the evidence for RDV therapy in mild-COVID-19 in real-world settings, especially in immunocompromised patients, is scarce.

We assessed the effectiveness and safety of RDV in high-risk outpatients with mild-to-moderate COVID-19 and whether immunocompetence status could influence the outcome.

## 2 Methods

This is a retrospective real-world study conducted in two hospitals in Spain (Dr. Balmis General University Hospital (HGUA) and Vega Baja Hospital of Orihuela (HVB), Alicante). We included adult patients with mild-to-moderate SARS-CoV-2 infection (confirmed by RT-PCR) at high risk for disease progression, who were treated as outpatients with a 3-day course of RDV, from 1st January to 30th September 2022.

### 2.1 Eligibility criteria for treatment

Treatment was initiated according to the treatment criteria established by The Spanish Agency for Medicine and Health Products (AEMPS) during the study period. Treatment was initiated in outpatients with mild-to-moderate infection in the first 7 days of symptom onset and stratified into one of the following categories: 1) all unvaccinated patients aged >65 years; 2) immunocompromised (IC) patients; and 3) patients aged >65 years, vaccinated >180 days after the last dose with at least one of the following risk factors for disease progression: diabetes mellitus, with complications, obesity (body mass index >35 kg/m^2^), chronic pulmonary disease, neurocognitive disorders and chronic cardiovascular and renal diseases.

The implementation of the clinical pathway and management protocol has been previously reported (Pinargote-Celorio et al., 2022). No patient had received oral treatment prior to the administration of remdesivir.

### 2.2 Variables and data collection

The demographic and clinical data at HGUA were obtained from the structured clinical registry developed using the *AppSheet®* platform (a no-code development platform by Google, LLC) for data management. The variables at HVB were extracted from electronic medical records. In both hospitals, the variables were recorded by expert clinicians, thereby minimizing information bias. The main explanatory variable was immunocompetence status. All patients were followed for a minimum of 30 days.

### 2.3 Outcomes

The primary effectiveness endpoint was a composite of any cause of hospitalization or death by day 30 (excluding those produced in the first 24 h of starting treatment). The cause of hospitalization and mortality was registered. The safety endpoint was any adverse event.

### 2.4 Statistical analysis

Categorical variables are expressed as frequencies and their percentages (%). Continuous variables are expressed through means and standard deviations or medians and interquartile range (IQR), depending on whether or not they follow a normal distribution. The index date was the outpatients’ COVID-19 clinical assessment date, and the final follow-up date was 30 days later, unless previously censored.

Multiple logistic regression models were built to explore risk factors associated with hospitalization or fatal outcome; adjusted odds ratios (aOR) with 95% confidence intervals (95% CI) were estimated. Variables were included as covariates if they showed associations in simple models with *p*-values below 0.100 and were considered main confounding factors such as age [≤80/>80], gender [male/female], and IC status [IC/non-IC]).

The statistical analysis of the results was carried out using the IBM SPSS Statistics version 22 program (IBM Corp., Armonk, NY). A *p* < 0.050 was defined as statistically significant.

## 3 Results

A total of 393 patients were treated during the study period, of which 211 patients were included in the analysis (flowchart, Supplementary Figure Sl). The basal demographic characteristics, comorbidities, and outcomes are shown in Table 1. The median age of patients was 65 years (IQR: 53–77), of which 54.0% were males and 70.1% were vaccinated with one booster. A total of 129 (61.1%) patients were IC and 38% had more than two comorbidities. The median duration of symptoms before first RDV infusion was 3 days (IQR 2–5).

**TABLE 1:** Basal demographic characteristics and comorbidities in the global cohort and based on evolution.

Variable	Overall (*N* = 211)	Hospitalization or death (*N* = 14)	Non-hospitalization or death (*N* = 197)	*p*-value
Sex, male, n (%)	114 (54.0)	11 (78.6)	103 (52.3)	0.057
Age, year, median (IQR)	65 (53–77)	70.5 (61–84)	65 (53–76)	0.258
**Refer to the clinic by,** n (%)				
Automatic screening	59 (28.0)	4 (28.6)	55 (27.9)	0.958
Emergency department	54 (25.6)	5 (35)	49 (24.9)	0.369
Primary care	53 (25.1)	1 (7.1)	197 (24.6)	0.108
Other services	45 (21.3)	4 (28.6)	41 (20.8)	0.463
**SARS-CoV-2 immunity status,** n (%)				
Two or less doses of vaccination	24 (11.7)	2 (14.3)	22 (11.5)	0.750
Three or more doses of vaccination	182 (88.3)	170 (88.5)	12 (85.7)	0.750
Previous SARS-CoV-2 infection	9 (4.3)	0	9 (4.6)	0.414
Days between vaccination infection and median (IQR)	134 (88–180)	109 (53–170)	134 (91–180)	0.325
**Comorbidities**, n (%)				
Smoking	70 (33.2)	8 (57.1)	62 (31.5)	0.074
Hypertension	129 (61.1)	9 (64.3)	120 (60.9)	0.803
Diabetes mellitus	62 (29.4)	4 (28.6)	58 (29.4)	0.945
Obesity (BMI>35)	61 (28.9)	3 (21.4)	58 (29.4)	0.762
Cardiovascular disease	49 (23.2)	5 (35.7)	44 (22.3)	0.252
Chronic lung diseases	26 (12.3)	3 (21.4)	23 (11.7)	0.389
eGFR <30 mL/min/m^2^	37 (17.5)	2 (14.3)	35 (17.8)	0.741
**Number or risk factors for severe COVID-19** ^a^, n (%)				
0	3 (1.49)	1 (7.1)	2 (1.0)	0.187
1	129 (61.1)	9 (64.3)	120 (60.9)	0.871
2	2 (0.9)	0 (0.0)	2 (1.0)	0.999
3	65 (30.8)	4 (28.6)	61 (31.0)	0.875
4	12 (5.7)	0 (0.0)	12 (6.1)	0.999
**Criteria of treatment**, n (%)				
Immunocompromised	129 (61.1)	9 (64.3)	120 (60.9)	0.803
Anti-CD20 therapy	16 (7.6)	3 (21.4)	13 (6.6)	0.071
Solid organ transplant	27 (12.8)	1 (7.1)	26 (13.2)	0.999
Anti-TNF therapy	20 (9.5)	1 (7.1)	19 (9.1)	0.999
Ongoing chemotherapy	19 (9.0)	2 (14.3)	17 (8.6)	0.366
Age>65 years, vaccinated >6 months, and at least one comorbidity	61 (28.9)	4 (28.6)	57 (28.9)	0.999
Other	17 (8.1)	16 (8.1)	1 (7.1)	0.999
Not vaccinated	4 (1.9)	0 (0.0)	4 (2.0)	0.999
**Time to treatment**				
Days since the first symptom to RDV, median (IQR)	3 (2–5)	5 (3–7)	3 (2–5)	0.058
More than 4 days since the first symptoms to RDV, n (%)	67 (31.8)	8 (57.1)	59 (29.9)	**0.035**
**Tolerance of treatment**, n (%)				
Serious adverse events	2 (0.9)	1 (7.1)	1 (0.5)	0.129
Discontinuation RDV for AE	1 (0.5)	1 (7.1)	0 (0.0)	0.066

AE, adverse events; BMI, body mass index; RDV, remdesivir; TNF, tumor necrosis factor; eGRF, estimated glomerular filtration rate.

^a^ Risk factors: two or less doses of vaccination, diabetes mellitus, obesity, cardiovascular disease, lung or renal chronic disease, and immunosuppression.

In bold, *p* < 0.05.

In the IC subpopulation, the main underlying conditions were as follows: 27 (20.9%) underwent solid organ transplant, 20 (15.5%) and 19 (14.7%) had ongoing chemotherapy for cancer, 16 (12.4%) underwent anti-CD20 therapy, 16 (12.4%) were given other biologic immunosuppressive drugs, 10 (7.0%) were given non-biological immunosuppressive drugs, and 21 (16.3%) had other immunosuppressive conditions (Table 2).

**TABLE 2:** Basal demographic characteristics and comorbidities based on the immunocompromised status.

Variable	Immunocompromised (*N* = 129)	Non-immunocompromised (*N* = 82)	*p*-value
Sex, male, n (%)	66 (51.2)	48 (58.5)	0.295
Age, year, median (IQR)	59 (46–67)	77 (70–849	**<0.001**
**Refer to the clinic by,** n (%)			
Automatic screening	39 (30.9)	20 (24.4)	0.357
Emergency department	26 (20.6)	28 (34.1)	**0.023**
Primary care	32 (24.8)	21 (25.6)	0.896
Other services	32 (24.8)	13 (15.9)	0.122
**SARS-CoV-2 immunity status,** n (%)			
Two or less doses of vaccination	16 (12.6)	8 (101)	0.591
Three or more doses of vaccination	111 **(7.8)**	71 **(3.9)**	0.591
Previous SARS-CoV-2 infection	8 (6.2)	1 (1.29)	0.158
Days between vaccination-infection, median (IQR)	128 (79–177)	152 (112–187)	**0.023**
**Comorbidities**, n (%)			
History of smoking	43 (33.3)	27 (32.9)	0.951
Hypertension	66 (51.2)	63 (76.8)	**<0.001**
Diabetes mellitus	30 (23.3)	32 (39.0)	**0.014**
Obesity (BMI>35)	27 (20.9)	34 (41.5)	**<0.001**
Cardiovascular disease	7 (5.4)	14 (17.1)	**0.006**
Chronic lung diseases	10 (7.8)	16 (19.5)	**0.011**
eGFR <30 mL/min/m^2^	9 (7.0)	28 (34.1)	**<0.001**
**Number or risk factors for severe COVID-19** ^a^, n (%)			
0	0 (0.0)	3 (3.7)	0.057
1	125 (96.9)	4 (4.9)	**<0.001**
2	0 (0.0)	2 (2.49	0.150
3	1 (0.8)	64 (78.0)	**<0.001**
4	3 (2.3)	9 (11.0)	**0.013**
**Time to treatment**			
Days since the first symptom to RDV, median (IQR)	4 (2–5)	3 (2–4)	0.102
More than 4 days since the first symptom to RDV, n (%)	47 (36.7)	20 (24.6)	0.067
**Tolerance of treatment**, n (%)			
Serious AE^b^	2 (1.6)	0 (0.0)	0.523
Discontinuation RDV for AE	1 (0.8)	0 (0.0)	0.999
**Outcome (30 days)**			
All-cause			
Hospitalization, n (%)	9 (7.0)	5 (6.1)	0.999
Days from treatment to admission, median (IQR) (*n* = 14)	17 (13–26)	2 (0–6)	**0.007**
Mortality, n (%)	0	2 (2.4)	0.150
COVID-19-related			
Hospitalization, n (%)	4 (3.1)	2 (2.4)	0.999
Days from treatment to admission, median (IQR) (*n* = 6)	10.5 (6.5–15.0)	1 (0.0–2.0)	0.133
Mortality, n (%)	0	0	-

Abbreviation: AE, adverse events; BMI, body mass index; eGRF, estimated glomerular filtration rate; IQR, interquartile range; RDV, remdesivir.

^a^ Risk factors: two or less doses of vaccination, diabetes mellitus, obesity, cardiovascular disease, lung or renal chronic disease, and immunosuppression.

^b^ Adverse events: One hepatic toxicity and one gastrointestinal intolerance.

In bold, *p* < 0.05.

### 3.1 Clinical outcomes and associated factors

The primary effectiveness endpoint was met by 196 patients. Fourteen (6.6%) patients were hospitalized and two died (0.9%) (the deaths of both patients were considered unrelated to COVID-19). Six patients (2.8%) were admitted for COVID-19-related pneumonia. No one required mechanical ventilation.

Nine of 129 patients (7%) in the IC group and five of 82 (6.1%) in the non-IC group had been hospitalized by day 30 (p, non-significant); COVID-19-related hospitalization rates were 3.1% and 2.4%, respectively (p, non-significant).

Multivariate analysis included the variables with a *p*-value < 0.100 (treatment with anti-CD20, days since first symptoms to RDV more than 4 days, and history of smoking), gender, age, and IC status. Treatment with anti-CD20 was independently associated with the combined outcome (hospitalization and death) [aOR 5.35 (95% CI 1.02–27.5); *p* = 0.047], whereas a duration of more than 4 days from symptom onset to RDV infusion [aOR 3.11 (95%CI 0.99–10.55), *p* = 0.050] was close to signification (Table 3). Finally, the immunocompetence status was not associated with hospitalization or death [aOR 1.15 (95% CI 0.37–3.57); *p* = 0.771]. When analyzing non-death hospitalization cases, the multivariate analysis included the variables with a *p*-value < 0.100 (treatment with anti-CD20 and days since first symptoms to RDV more than 4 days), gender, age, and IC status. Treatment with anti-CD20 was not associated with non-death hospitalization (Table 4).

**TABLE 3:** Crude and adjusted odds ratios of hospitalization with or without death (14) vs. non-death non-hospitalization (*n* = 197).

Variable	Crude OR (95% CI)	*p*-value	Adjusted OR (95% CI) *	*p*-value
Sex, male, n (%)	3.34 (0.01–12.36)	0.057	3.03 (0.74–12.34)	0.122
Age >80	2.14 (0.63–7.28)	0.258	3.44 (0.73–16.25)	0.118
**Refer to the clinic by,** n (%)				
Automatic screening	1.03 (0.31–3.43)	0.958	NI	
Emergency department	1.67 (0.53–5.24)	0.369	NI	
Primary care	0.21 (0.03–1.68)	0.108	NI	
Other services	1.52 (0.54–5.10)	0.463	NI	
**SARS-CoV-2 immunity status,** n (%)				
Two or less doses of vaccination	1	0.750	NI	
Three or more doses of vaccination	0.77 (0.16–3.70)	0.750	NI	
Previous SARS-CoV-2 infection	NA	0.414	NI	
Days between vaccination infection, median (IQR)	0.99 (0.99–1.00)	0.325	NI	
**Comorbidities**, n (%)				
Smoking	2.90 (0.96–8.72)	0.074	2,69 (0.80–9.03)	0.109
Hypertension	1.15 (0.37–3.57)	0.803	NI	
Diabetes mellitus	0.96 (028–3.18)	0.945	Ni	
Obesity (BMI>35)	0.65 (0.17–2.43)	0.762	NI	
Cardiovascular disease	1.93 (0.61–6.06)	0.252	NI	
Chronic lung diseases	2.06 (0.53–7.98)	0.389	NI	
eGFR <30 mL/min/m^2^	0.77–0.16–3.60)	0.741	NI	
**Number or risk factors for severe COVID-19** ^a^, n (%)				
0	7.50 (0.63–88.24)	0.187	NI	
1	1.15 (0.37–3.57)	0.871	NI	
2	NA	0.999	NI	
3	0.89 (0.29–2.95)	0.875	Ni	
4	NA	0.999	NI	
**Criteria of treatment**, n (%)				
Immunocompromised	1.15 (0.37–3.57)	0.803	1.15 (0.37–3.57)	0.771
Anti-CD20 therapy	3.86 (0.95–15.57)	0.071	5.35 (1.02–27.52)	0.047
Solid organ transplant	0.50 (0.06–4.03)	0.999	NI	
Anti TNF therapy	0.72 (0.09–5.81)	0.999	NI	
Ongoing chemotherapy	1,76 (0.36–8.54)	0.366	NI	
Age>65 years, vaccinated >6 months and at least, one comorbidity	0.98 (0.29–3.26)	0.999	NI	
Other	0.87 (0.10–7.09)	0.999	NI	
Not vaccinated	NA	0.999	NI	
**Time to treatment**				
More than 4 days since the first symptom to RDV, n (%)	**3.11 (1.03–9.38)**	**0.035**	**3.11 (0.99–10.55)**	**0.050**
**Tolerance of treatment**, n (%)				
Serious adverse events	15.07 (0.89–254)	0.129	NI	
Discontinuation of RDV for AE	NA	0.066	NI	

Abbreviation: NA, not available; NI, not included,

^a^ Made by multiple logistic regression model. Variables were included as covariates if they showed associations in simple models with *p*-values below 0.100 and were considered main confounders such as age [≤80/>80], gender [male/female], and immunocompromised status [immunocompromised/non-immunocompromised].

In bold, *p* < 0.05.

**TABLE 4:** Crude and adjusted odds ratios of with or without death (*n* = 12) vs. non-death non-hospitalization (*n* = 197).

Variable	Crude OR (95% CI)	*p*-value	Adjusted OR (95% CI)^a^	*p*-value
Sex, male	2.73 (0.72–10.41)	0.126	3.41 (0.84–13.78)	0.085
Age >80	1.07 (0.22–5.12)	0.999	1.98 (0.30–12.74)	0.472
**Refer to the clinic by**				
Automatic screening	0.86 (0.22–3.29)	0.999	NI	
Emergency department	2.15 (0.65–7.18)	0.129	NI	
Primary care	0.25 (0.03–2,21)	0.303	NI	
Other services	1.27 (0.32–4.89)	0.719	NI	
**SARS-CoV-2 immunity status**				
Two or less doses of vaccination	1	0.999	NI	
Three or more doses of vaccination	1.43 (0.17–11–56)	0.999	NI	
Previous SARS-CoV-2 infection	NA	0.414	NI	
Days between vaccination infection	0.99 (0.98–1.00)	0.136	NI	
**Comorbidities**				
Smoking	2.17 (0.67–7.02)	0.211	NI	
Hypertension	1.28 (0.37–4.40)	0.770	NI	
Diabetes mellitus	0.79 (0.21–3.06)	0.999	Ni	
Obesity (BMI>35)	0.48 (0.10–2.25)	0.515	NI	
Cardiovascular disease	1.73 (0.50–6.04)	0.477	NI	
Chronic lung diseases	2.06 (0.53–7.98)	0.389	NI	
eGFR <30 mL/min/m^2^	0.42 (0.05–3.36)	0.696	NI	
**Number or risk factors for severe COVID-19**				
0	NA	0.999	NI	
1	1.92 (0.50–7.33)	0.3.79	NI	
2	NA	0.999	NI	
3	0.74 (0.19–2.84)	0.759	Ni	
4	NA	0.999	NI	
**Criteria of treatment**				
Immunocompromised	1.93 (0.50–7.33)	0.379	1.63 (0.32–8.26)	0.553
Anti-CD20 therapy	4.71 (1.14–19.56)	0.053	4.23 (0.86–20.92)	0.077
Solid organ transplant	0.59 (0.07–4.82)	0.999	NI	
Anti-TNF therapy	0.82 (0.10–6.96)	0.999	NI	
Ongoing chemotherapy	2.11 (0.43–10.46)	0.299	NI	
Age>65 years, vaccinated >6 months and at least, one comorbidity	0.49 (0.10–2.31)	0.516	NI	
Other	1.03 (0.13–8.48)	0.999	NI	
Not vaccinated	NA	0.999	NI	
**Time to treatment**				
More than 4 days since the first symptom to RDV	**2.27 (0.99–10.73)**	**0.054**	2.55 (0.52–10.45)	0.248
**Tolerance of treatment**				
Serious adverse events	17.81 (1.04–304)	0.122	NI	
Discontinuation RDV for AE	NA	0.057	NI	

Abbreviations: NA, not available; NI, not included.

^a^ Made by the multiple logistic regression model. Variables were included as covariates if they showed associations in simple models with *p*-values below 0.100 and were considered main confounders such as age [≤80/>80], gender [male/female] and immunocompromised status [immunocompromised/non-immunocompromised].

In bold, *p* < 0.05.

### 3.2 Safety

Two patients presented moderate toxicity, with discontinuation due to gastrointestinal intolerance in one patient after the second dose of RDV.

## 4 Discussion

Among vaccinated outpatients with mild-to-moderate COVID-19 and very high risk of disease progression, treatment with a 3-day course of RDV was associated with a low rate of hospitalization or death for any cause (less than 7%) during the Omicron variant wave. The immunocompetence status, as a global risk factor, does not seem to influence the response to RDV treatment. Only treatment with anti-CD20 was independently associated with hospitalization and death in this population treated early with RDV.

Both vaccination and the Omicron variant have been associated with a more benign course, with hospitalization rates, without treatment, of 0.5%–3.2% (in large published cohort studies), (Arbel et al., 2022; Najjar-Debbiny et al., 2022; Aggarwal et al., 2023; Butler et al., 2023), but these reports included younger populations with less comorbidity than in our study.

Evidence for RDV therapy in improving clinical outcomes in non-hospitalized patients with moderate-to-severe COVID-19 is scarce. The rates of hospitalization or death in the only outsetting clinical trial of RDV (PINETREE), (Gottlieb et al., 2022) were 0.7% for RDV vs. 5.3% for placebo [HR 0.13 (95% CI 0.03–0.59)]; however, this study only included non-vaccinated population, with very few immunosuppressed patients (5%) during the wave not pertaining to Omicron.

Recently, several small observational studies of RDV treatment for IC outpatients have been published by Rajme-López et al. (2022), who in a retrospective cohort of IC patients (*N* = 126) reported a significant reduction in hospitalization or death [aHR 0.16 (95% CI 0.06–0.44)]. Piccicacco et al. (2022) showed that among 260 patients (73.3% IC), those treated with RDV or sotrovimab had lower likelihoods of hospitalization than matched high-risk control patients (11% vs. 8% vs. 23.3%, respectively). Finally, Solera et al., (2023) reported a lower hospitalization rate (2.3% vs. 12.3%) in a cohort of solid organ transplant recipients treated with RDV vs. controls. Hospitalization rates in these reports ranged from 2.3% to 11% similar to those of 6% patients in our cohort.

In these reports, hospitalization for any cause has been used as the main outcome. However, in patients with complex medical disorders, hospitalizations related to underlying condition, and not to COVID-19, are common, hindering the interpretation of the results. A definition based on the number of COVID-19-related hospitalizations or death would be better to assess the effectiveness of treatment. In our cohort, the COVID-19-related hospitalization rate was less than 3%, and no deaths were registered. Although the patients in the IC cohort were younger and vaccinated more frequently, the non-IC group had a higher number of comorbidities; however, the rate of progression was similar after adjusting for confounding factors. Hence, based on our results, early RDV treatment could be as effective in IC patients as in non-IC patients with other high-risk factors.

Administration of monoclonal anti-CD20 antibody has been associated with worse clinical outcomes in patients with COVID-19 (Calderón-Parra et al., 2022). In our cohort, anti-CD20 was the only risk factor associated with less clinical benefit of early 3-day treatment with RDV. We, therefore, think that these patients may require a different approach, with a longer duration of treatment or combination of drugs. Our data, close to statistical significance, support the importance of early RDV treatment. In some settings, daily IV administration of remdesivir for 3 days may be a logistical challenge and increase the cost compared to oral treatment. However, for patients with limitations for treatment with nirmatrelvir/ritonavir (renal insufficiency or severe pharmacokinetic interactions), it may be the only available alternative.

Some important limitations need to be addressed. First, this was a two-center retrospective observational study, and although an effort was made to control for relevant confounding factors, unmeasured confounding variables may still have been present. Second, this study has no control group (it would have been unethical not to administer treatment according to the 2022 COVID-19 guidelines to very high-risk patients). Finally, viral sequencing was not systematically performed; we assume an Omicron variant of SARS-CoV-2 as the dominant circulating strain based on data tracking reported by our center. These limitations were counterbalanced by a large cohort of high-risk patients managed through a clinical pathway, allowing for a comparative analysis of outcomes.

## 5 Conclusion

In this outpatient real-world study during the Omicron wave, early treatment with a 3-day course of remdesivir had a good safety profile and prevented disease progression regardless of immunocompetence status. These data support another therapeutic option for the management of non-hospitalized vulnerable vaccinated patients who are at high risk for progression to severe COVID-19.

## Data Availability

The raw data supporting the conclusion of this article will be made available by the authors, without undue reservation.
