# Supplementary figures and images for: A retrospective real-world study of early short-course remdesivir in non-hospitalized COVID-19 patients at high risk for progression: low rate of hospitalization or death, regardless of immunocompetence status
Source: Front Pharmacol. 2023 Oct 10;14:1218650. doi: 10.3389/fphar.2023.1218650 (PMC10597661; doi:10.3389/fphar.2023.1218650)

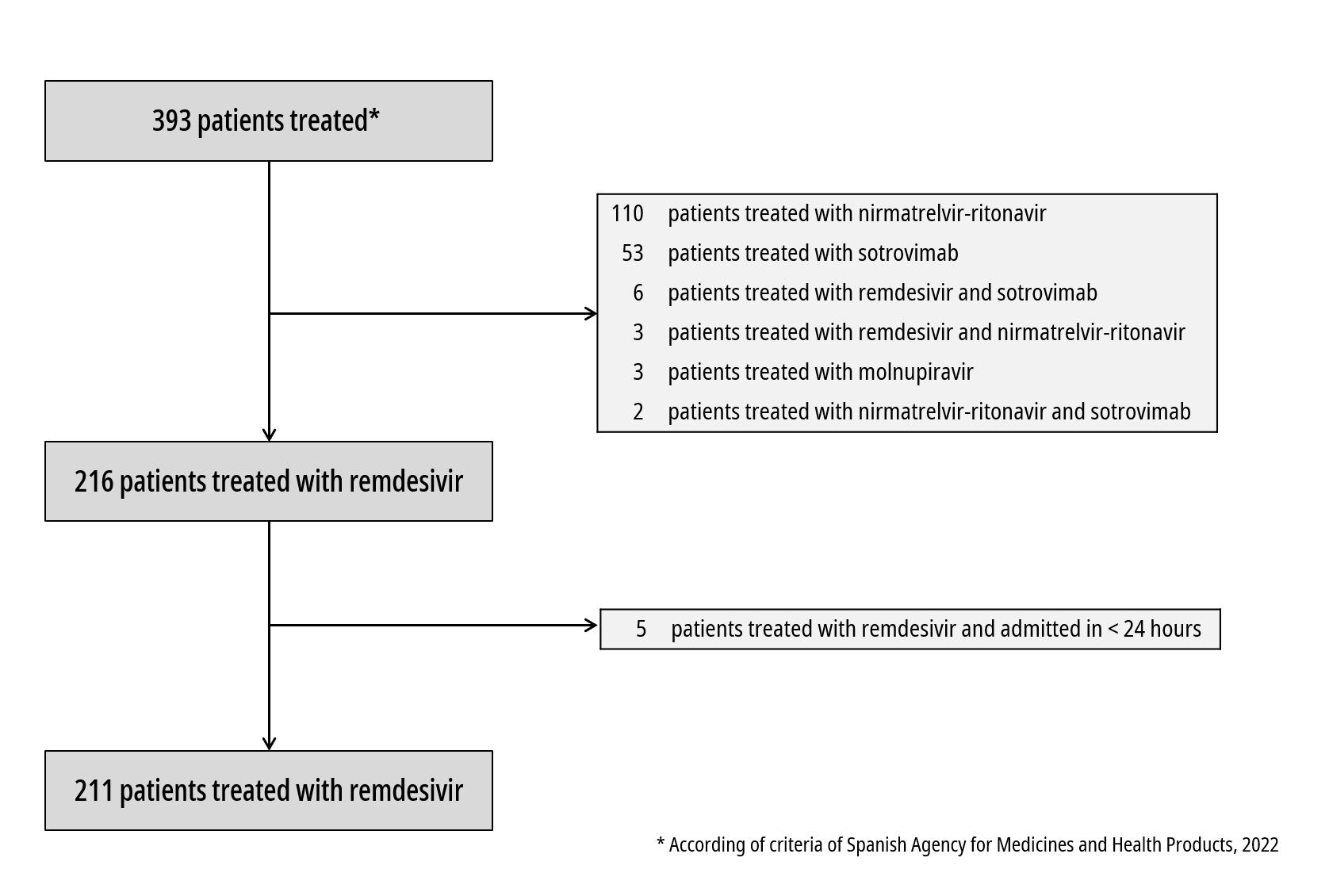

Supplement: Supplementary file 2 [file Image1.tif]
